# Implementation and adaptation of a hub-based psychiatric and primary care program: A qualitative descriptive analysis of The Seamless Care Optimizing the Patient Experience (SCOPE) Mental Health program

**DOI:** 10.1371/journal.pone.0303750

**Published:** 2024-05-28

**Authors:** Carly Whitmore, Mona Emam, Pauline Pariser, Blanca Bolea

**Affiliations:** 1 School of Nursing, McMaster University—Hamilton, Ontario, Canada; 2 Centre for Addiction and Mental Health—Toronto, Ontario, Canada; 3 Women’s College Hospital, Toronto, Ontario, Canada; 4 Seamless Care Optimizing the Patient Experience (SCOPE) Mental Heath Program, Canada; 5 SCOPE Program, University Health Network, Toronto, Ontario, Canada; Hosei University: Hosei Daigaku, JAPAN

## Abstract

**Background:**

The Seamless Care Optimizing the Patient Experience (SCOPE)–Mental Health program is a comprehensive case management and psychiatric care initiative that supports primary care physicians in independent medical practices. This program offers a range of services that aims to enhance primary care capacity for mental health and provide accessible clinical care for patients. With its flexible hub-based approach, this program allows participating sites to tailor their implementation based on their available resources and specific needs within their community.

**Objectives:**

The aim of this quality improvement initiative was to investigate the evolution of this collaborative mental health model, focusing on specific site adaptations, local implementation challenges, and opportunities for ongoing development and sustainability across SCOPE sites in the Greater Toronto Area.

**Method:**

This evaluation employed a qualitative descriptive design where semi-structured interviews, guided by the Reach Effectiveness Adoption, Implementation, and Maintenance framework were conducted with staff from all 8 SCOPE-Mental Health sites. Site representatives were interviewed virtually between March and July 2023 and data were analyzed using qualitative content analysis.

**Findings:**

The SCOPE-Mental Health model permits flexibility through specific local adaptations led by community need that leverage existing assets either at the site or within the individual community. Adoption by primary care physicians was crucial to program success and facilitated efficiency and interprofessional collaboration. Maintenance efforts included pathway refinement, and marketing and funding considerations. Challenges to program development included continuity of staff, physician compensation issues, and electronic health record interoperability. The SCOPE-Mental Health program fosters linkages among unaffiliated primary care offices, hospitals, and community-based resources to improve mental health care. Key recommendations include advocating for sustainable funding and facilitated mechanisms for psychiatric consultations.

**Conclusions:**

This initiative offers valuable insights for healthcare organizations seeking to develop similar programs, emphasizing the need for tailored approaches and ongoing evaluation to ensure a lasting impact in underserved communities.

## Introduction

The Seamless Care Optimizing the Patient Experience (SCOPE) program is a comprehensive healthcare initiative designed to enhance the overall experience and continuity of care for patients across various healthcare settings [1, 2]. Developed to address the fragmentation and inefficiencies often observed in healthcare systems, the key features of the SCOPE program include: care integration, patient-centered care, care continuity, integration of technology, patient navigation, access to resources, patient and provider education, and quality improvement to fuel scalability and expansion. While the SCOPE program is dedicated to enhancing patient care across diverse medical conditions and populations, [3] there was a recognized need to support primary care providers (PCPs) in providing comprehensive mental health care within the broader SCOPE population. In response, the SCOPE Mental Health program was developed [4].

### SCOPE Mental Health

The SCOPE Mental Health (SCOPE-MH) initiative is an integrative case management and psychiatric care program, delivered in a hub-based model, and dedicated to supporting Primary Care Physicians (PCPs) who are operating in unaffiliated medical practices. Aligned to the Quintuple Aim for Healthcare Improvement (patient experience, population health, reducing costs, care team well-being, and health equity), [5] the SCOPE-MH program is designed to deliver a range of services, including patient resource guidance, social support, counseling, psychiatric consultation, and short-term follow-up care. SCOPE-MH operates with funding allocations to hospitals, allowing each participating site the discretion to tailor its implementation of the model. This flexibility extends to the allocation of resources, including the staffing complement, as well as varying levels of psychiatric services across sites. For example, some sites have elected to use funding to employ a nurse navigator to screen and coordinate care, while others have funded a social worker as the first point of contact.

The SCOPE-MH program is an innovative approach to build capacity in primary care and address lengthy waiting lists and connect primary care to psychiatric specialty to tackle the mental health needs of individuals within the community [6]. Through this collaborative care program, PCPs are supported and accompanied through the patient’s mental health care journey, exposing PCPs to new resources, treatment modalities and options, and, ultimately, increasing comfort and confidence with treating more complex patients. SCOPE-MH is a low barrier, easy to access program as PCPs do not have to complete lengthy referrals and patients can directly access care via phone, email, or web-conferencing without a referral, so long as their PCP is registered with the service. Further, a new referral is not required if a patient has previously accessed the service and after a few months or years have a new need. Contact with the program, typically initiated by a social worker, is designed to occur within 72 hours with care delivered flexibly, often entirely virtually.

### SCOPE-MH pilot and adaptations

**Fig 1:**
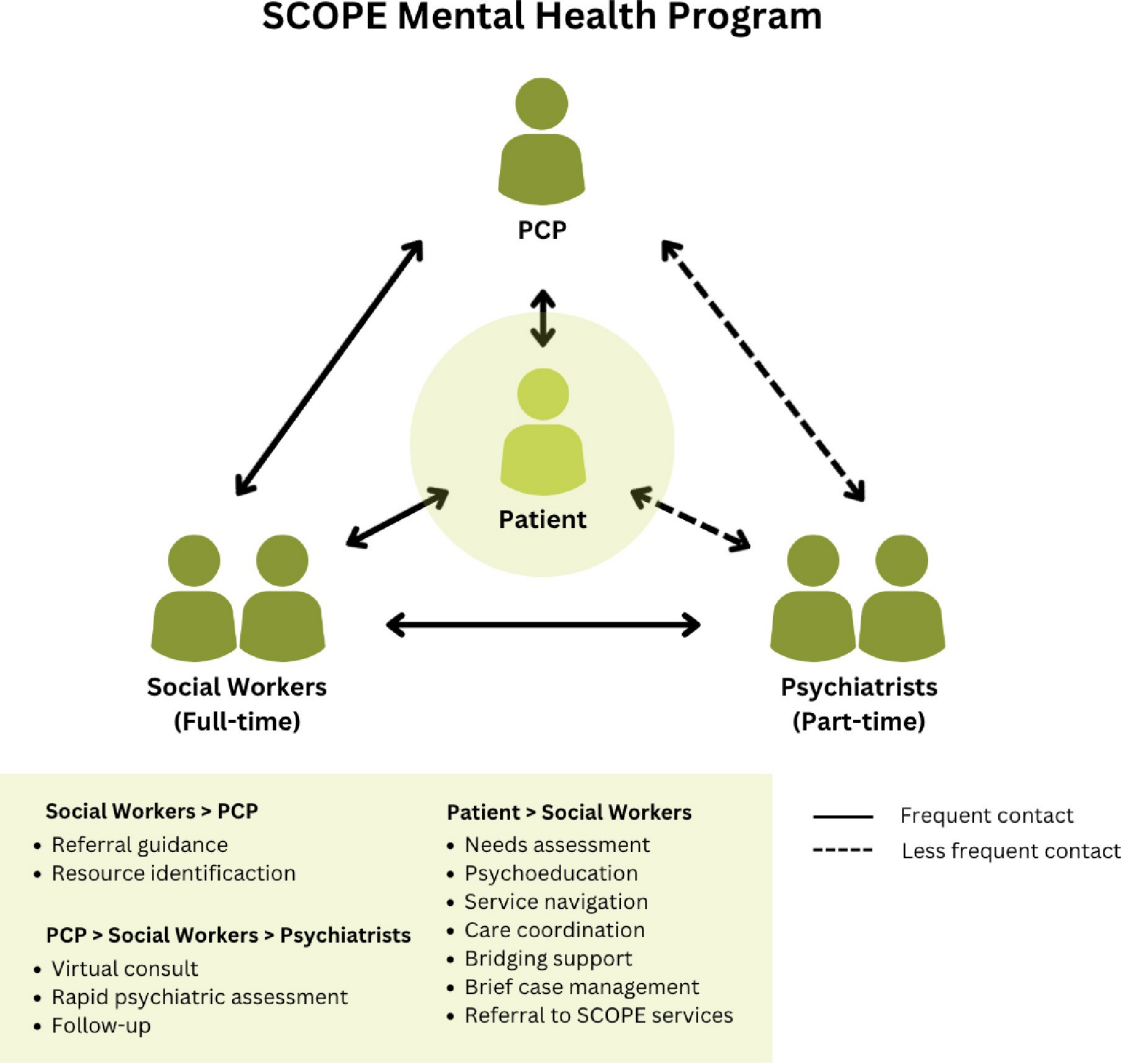
Initial SCOPE-Mental Health model.

SCOPE-MH was piloted in a mid-sized ambulatory hospital in Mid-West Toronto. The initial model (see Fig 1) was based on the Collaborative Care Model [7, 8]. This model facilitates easy access to care by situating a behavioral health manager as the first point of care. The behavioral health manager provides counselling and social support, coordinates with specialists (e.g., psychiatrist), and communicates directly with PCPs. In the pilot for SCOPE-MH, experienced social workers acted as behavioral health managers. Care was designed to be patient-driven, with differing levels of care provided by the same team according to patient identified needs. This pilot site is now a full-fledged service providing care to over 850 patients per year, from over 100 PCPs, in a largely virtual model. This includes two full-time social workers, two part-time psychiatrists, and a part-time administrative support person. At this site, most of these practices are unaffiliated with team-based allied health staff, often serving ethnic and linguistic minority populations living in the city.

Mentored by this initial pilot program, eight further sites have developed across the greater Toronto area. Each of these sites has adapted the initial model to best serve their individual community needs. For example, a hospital and outpatient health organization serving several communities in suburban Toronto has adapted this model to offer a nurse-led service, where PCPs can call directly and, depending on need, be connected either to an urgent mental health clinic to receive mental health navigation services, or speak directly to a psychiatrist. In a rural pilot site, a social worker acts as a behavioural health manager and offers brief counselling, waitlist relief for a local outpatient mental health service, and resource navigation.

Reflective of the sociocultural diversity of the greater Toronto area, SCOPE-MH is an equitable intervention that focuses on family physicians that often work with ethnic or linguistic minorities which often experience greater challenges to find and access specialized healthcare [9]. Patients may access the program several times and with different care needs without having to see a physician if they do not wish to. They do not have to repeat their health history to different healthcare professionals and they are granted time to reflect on options of care knowing that they can contact their navigator at any time during the process.

Due in part to the complexity of mental health, the need for long-term follow-up, and ongoing issues with system fragmentation, it is uncommon to evaluate mental health models of care. However, considering the innovative patient-centered allied health delivered program responding to the full bio-psycho-social model of health and wellness, there is a need to understand not only the impact of this program, but also the sustainability of the initiative. In evaluating the SCOPE-MH program, there is an opportunity to contribute valuable insights to the broader field of mental health service delivery and primary care integration. The purpose of this quality improvement initiative was to better understand the development of this collaborative mental health care model with particular interest on site adaptations, explore the local challenges of implementing the SCOPE-MH program, and identify opportunities for further development and sustainability across SCOPE sites in the greater Toronto area.

## Methods

A qualitative descriptive design was used to evaluate the development of SCOPE-MH across all participating sites and understand challenges and opportunities. The purpose of using a qualitative descriptive design was to generate practical insights and actionable recommendations to inform future development and refinement of the program. Detailed information was collected directly from interviews with SCOPE-MH team members. Data generation and analysis were both guided by the Reach, Effectiveness, Adoption, Implementation, and Maintenance (RE-AIM) framework, designed to improve transparency in reporting the essential components of a program to support adoption and implementation of evidence-based interventions.

The RE-AIM framework is frequently used as a planning tool for sustaining the spread of interventions [10]. In considering all five domains, areas of improvement can be identified to maximize the potential impact of an intervention. Project methods and evaluation findings follow the Standards for Reporting Qualitative Research [11].

### Participants

For this evaluation project, participants included interdisciplinary staff members as representatives from each of the eight SCOPE-MH hub sites. An invitation from a research assistant (ME) was sent to each site on March 2, 2023. This invitation included a purpose statement for the evaluation and potential dates for the interview. Each site self-identified interdisciplinary team members to participate in the evaluation and there were no exclusion criteria for participation. The last interview was completed on June 23, 2023.

### Data generation

#### Interviews

All SCOPE-MH hub sites were invited to participate in a virtual semi-structured interview. These interviews were conducted by the evaluation team which included a Registered Nurse with specialized knowledge in psychiatric and mental health care and qualitative methodology (CW), a Research Assistant who has supported the implementation of SCOPE-MH and with prior experience in conducting and supporting qualitative studies (ME), and a Psychiatrist who is the director for the SCOPE-MH program (BBA) at the site that acted to germinate the program dissemination. Interviews were completed using web-conferencing software (Microsoft Teams) between March and June 2023. All interviews were digitally recorded and transcribed with transcriptions checked against the original audio for accuracy.

Interview questions were developed by the evaluation team and mapped to the RE-AIM framework. Acknowledging the varied roles that interdisciplinary team members have in delivering the SCOPE-MH program, alongside a set of core questions, interview guides were adapted to each professional role. For example, primary care practitioners were asked about their access to the SCOPE-MH program, psychiatrists were asked about factors that influence their decision to accept referrals through the program, nurses and social workers were asked about their needs in implementing and developing the program, and navigators were asked about the referral pathway for patients of the program. All evaluation participants were asked about how they viewed their involvement in SCOPE-MH as contributing to the aims identified in the Quintuple Aims, [12] as well as how they felt that they could contribute to further development of the SCOPE-MH program.

#### Data analysis

All anonymized interview transcripts and documents were uploaded to N.Vivo v12 for data management and analysis. Applying qualitative content analysis, a mix of both deductive coding, using the RE-AIM framework and set criteria and definitions of the framework, [13] and inductive analysis, was used. This analytical approach permitted an in-depth evaluation of the development, including adaptations of the program, across different sites as well as an identification of both challenges and opportunities for the implementation of the SCOPE-MH program. One evaluation team member (CW) independently analyzed all interview transcripts. Following this analysis, the full evaluation team (CW, ME, and BBA) met to review coding, identify relationships, discuss ideas, and select exemplar quotes to support established topic summaries.

#### Ethical considerations

As this project was for the purposes of quality improvement, and was confirmed as such by the SCOPE host site (Women’s College Hospital Research Ethics Board) prior to project start, no institutional ethics approval was sought. As part of the original SCOPE project design, ongoing evaluation of the service was planned. All participating team members verbally consented to recording the discussions. This consent was documented in the transcript.

### Findings

#### Participant characteristics

For this project, participants included interdisciplinary staff members from each of the eight SCOPE-MH sites across the greater Toronto area. A total of 29 team members were interviewed with 44% (n = 13) of the attendees identifying as Program, Clinic, or Project Managers, 31% (n = 9) as Social Workers or Nurses, 17% (n = 5) as Primary Care Providers or Leads, 6% (n = 2) as Psychiatrists. On average, three team members joined each interview. An overview of interview attendees from each site are detailed in Table 1.

**Table 1:** Interview attendees from the SCOPE-MH sites (n = 29).

Site	Interview Attendees			
	Program, Clinic, or Project Managers	Social Workers or Nurses	Primary Care Providers or Leads	Psychiatrists
**1**	X	X		
**2**	X	X	X	
**3**	XX	X	X	
**4**	XX	XX		
**5**	X	X	XX	XX
**6**	XXX	X		
**7**	XX	X		
**8**	X	X	X	

Note: Each X denotes one participant of that role who attended the interview.

#### SCOPE-MH development and implementation

Across all eight sites, the SCOPE-MH program has been fully implemented but with differing degrees of development. For some sites, the program has been operating for over a year, whereas with other sites, only a few months. Despite this difference, as well as differences in team membership and access to services due to geography, there were commonalities in how the model was developed, adapted, and in challenges and opportunities identified. Findings are reported and organized according to each of the components of the RE-AIM framework [13] with equity-related perspectives and considerations incorporated. Additionally, challenges to the uptake and implementation of SCOPE-MH are described.

*Reach*. Team members captured the reach of the SCOPE-MH program through their description of both the targeted population accessing the program, as well as in the uptake of the program at the level of the PCPs.

Common characteristics of patients who accessed the program included prevalent diagnostic needs and specific needs for care modalities. For example, team members across many of the sites identified adult attention deficit hyperactivity disorder as a prevalent referral reason for the program. The uptake of this specific condition reflects challenges in obtaining highly specialized psychiatric diagnoses in the community and emphasizes the need for both general and specialized psychiatric care. Beyond specific diagnoses, team members also identified that many of the patients who accessed the program had need for short-term therapy with reduced waiting lists. This included brief cognitive therapy, supportive therapy, and other counseling type services. By offering timely access to mental health services, the SCOPE-MH program was described as not only alleviating need, but also potentially mitigating the exacerbation of mental health conditions that may arise due to prolonged delays in care provision. However, in addition to these prevailing characteristics, most frequently, team members described that what was most common for those they supported through the SCOPE-MH program was instead paradoxically the “uncommon”.

“*I would just say the uncommon*. *Simply because the way I describe my role and the SCOPE program is that the usual process works fine most of the time for most doctors*, *but they’re reaching out to SCOPE for support when they’re stuck*. *Earlier this week I needed to find a dietitian that offers counseling in-person for a child with autism and some education about avoidant food disorder*. *That’s uncommon*. *I’ve never been asked that before*, *might never get asked that again*. *There’s many one-off requests like that and I spend a lot of time finding resources to support them*. *There really is not a whole lot that is common*.*”–Nurse Navigator*

In supporting the uncommon, the range of expertise across team members and the potential of the program to respond to the ever-evolving needs of complex patient needs and communities is exemplified. Demonstrating the patient-centeredness of the SCOPE-MH program, this ability to support complexity may contribute to the observed month-over-month increases in the number of PCPs accessing the program or the number of referrals made. Each of the SCOPE-MH sites described need for different services to fill different care gaps, and the varied ways that they approached these needs and gaps. For example, a more traditional uptake of the program involved the use of a social worker to navigate and coordinate care, while other sites identified more specific needs to address community-level gaps. Compared to other mental health models of care, this flexibility of how resources are utilized and what health professional roles are delivering the model is a unique facet of the SCOPE-MH program.

*Effectiveness*. While this evaluation did not explicitly measure effectiveness, across sites, team members described the impact of the SCOPE-MH program through the capacity and relationship building effect of the program. Expanding community capacity and relationships between PCPs, community supports, and other health adjacent professionals was described to occur continuously through engagement with the program. For example, team members identified that when a PCP would call to ask a specific question, they noted that the PCP would not contact them again when a similar need was identified:

“*It’s really iterative because*, *you know*, *once I’ve had that conversation with the psychiatrist*, *I’m not going to call in to ask that same question again in the future*.*”–PCP Lead*

Further supporting this capacity building finding is that several sites noted in interviews that while they may be engaging more PCPs, they were having fewer requests or referrals from existing connections. This signals that the relationships made available through the SCOPE-MH program can enhance skill and knowledge, potentially increasing the confidence of the PCP in addressing mental health issues. Similarly, it was noted that when resources were provided by the navigators, PCPs were quick to accept these resources, use them, and distribute them through their own networks. One navigator even reported later seeing resources provided to a PCP on their clinic website. This capacity and relationship building plays a pivotal role in strengthening the social fabric of the community. Team members identified that they felt that relationships were being forged where they had previously not existed, and that trust and interconnectedness were tangible bi-products of these relationships:

“*So much about these partnerships is relational*. *I think we were a bit surprised with how slow it was in the beginning*. *We were expecting it to be a flood of referrals right away but it makes sense that it takes time to get to know [PCPs] and build that trust*.*”–Social Worker*

*Adoption*. The uptake of the program by PCPs was described as pivotal to its success and to building momentum in the community. While there was an acknowledgement from team members that the program did not necessarily offer net-new resources, the program supported communities and PCP partners by creating efficiencies with existing resources. For many sites, this was evidenced by a steady increase in referrals being made and a consistent amount of time, including facetime, spent doing work for SCOPE-MH. While the team membership varied across sites, the expertise was consistently high and there were efforts being made to create communities of practice to support the teams. At one site, this expertise was described as a potential strategy to overcome their struggles with recruiting and retaining PCPs in their community:

“*What is also true is we have lots of [community name] residents with no primary care physician*. *Those physicians that are here are saturated*, *they are not accepting new patients*, *and then*, *because of that*, *they come to our emergency [department]*. *We think it could be a benefit for physicians to practice here if they do have more supports like those offered with SCOPE*. *I think there’s no harm marketing the SCOPE program on a larger scale*. *It would be appetizing for physicians and nurses*.*”–Program Manager*

At the individual settings, adoption was also demonstrated through the speed by which later sites were able to be up and running. For example, one of the sites was able to be fully operational within just 3 months. This was attributed to the mentorship, collaboration, and sharing across sites in addition to the expertise of the SCOPE team. This cross-site learning was facilitated through regular SCOPE team meetings where all sites shared progress and needs but was also described to have happened more organically. For example, one site described reaching out to a colleague at another site with questions and from this connection, a community of practice was established.

While adoption of the program was largely positive and demonstrated the scalability of SCOPE-MH, one challenge raised by team members was specific to a need to better promote, support, and encourage communication between PCPs and psychiatrists. For example, a recurring question from team members during interviews was about how PCPs could consult with psychiatrists. Across all sites, PCPs expressed a need to directly communicate with psychiatrists to have questions answered including those related to psychiatric medications, mental health forms, or ongoing follow-up, but not knowing how to initiate this or how this collaboration is compensated. These questions and concerns demonstrate a desire for intraprofessional collaboration, but highlight the limited support to do so in the existing health system.

*Implementation*. True to the spirit of the program, implementation of the SCOPE-MH program was tailored to the setting within its community. This included population- and community-specific adaptations, purpose-built interventions, and in the features of the program that were emphasized.

While each of the sites are geographically within the greater Toronto area, there is significant variation in demographics and in the availability and accessibility of services depending on site. For example, some sites interviewed are urban sites with populations that are highly ethnoculturally diverse. At these sites, team members described needing to find resources that were available in languages that are not the dominant ones. At other sites considered to be more rural, challenges with availability such as access to internet were present:

“*Sometimes it’s really just connection to reliable internet that is the tough part*. *There are a couple of folks who don’t have a computer*, *but they have a cell phone*, *but it’s a flip phone so I cannot send them e-mails*. *The technology piece is really challenging because I may have to ask them to drive to the town to access WiFi and that is not very private if they have to be in a library or something*.*”–Social Worker*

During these interviews, each site was able to clearly communicate the needs of their community, and the ways that they had purposefully adapted the SCOPE-MH program to fill gaps or address known challenges. For example, in one community, they had intentionally engaged youth and young adult supports while another site had recognized the extensive need for counseling in their community and, using SCOPE-MH funds, had contracted a private therapy clinic to provide wait-list relief. The ability of sites to adapt the program to fill their service needs using different features of the program was consistently described as a strength.

*Maintenance*. Delivering and sustaining the program over the long-term was top-of-mind for interviewed team members. This included strategies for marketing the program, consideration for the referral pathway, and careful thought specific to funding and staffing.

E-blasts, newsletters, and direct communication were all employed to recruit PCPs into SCOPE-MH. Team members described including testimonials, example resources, showcasing the low-barrier nature of the program, and even developing their own taglines:

“*The recent e-blasts were much more of a sort of marketing flyer*. *It had testimonials*, *it had a catchy title*. *After we sent it out*, *I don’t know*, *I think we had like five replies within the first half hour and about ten by the end of the week*.*”–Program Manager*

Team members described these marketing products and outreach attempts as not only bringing in new PCPs, but also spoke to the troubleshooting that occurred as a result of these conversations. For example, after one e-blast, one of the sites realized that in requiring their referral pathway to use a specific e-service provider, barriers to the program were created. Subsequent communication sought to clarify the pathway.

*Equity*. While not explicitly part of the RE-AIM framework, equity was a recurring theme identified in completed interviews and reflected throughout all discussion of the program. Despite the SCOPE-MH program not having been designed as a culturally-tailored program, the flexibility of the model has enabled sites to adapt the delivery of the care to community needs, including sites that were developed with input and feedback from community members and staff to adapt the program as well as sites that have added interpretation services and translated materials to ensure that language does not hinder access to mental health care

Across interviews, participants emphasized the ability of the SCOPE-MH program to enhance access to care. This included lowering the threshold to access (e.g., low barrier referrals or self-referral), bringing specialist services to less connected communities (i.e., distribution of resources), and better engaging existing community supports in an asset-based model (e.g., building collaborations with community-based organizations and other stakeholders to leverage resources). Aspirationally, in one of the interviews, a future state where SCOPE was able to eliminate barriers related to geography was described:

“*I think SCOPE can actually remove barriers that are territorial in some ways*. *Like*, *often*, *unless you live in a certain area*, *you cannot access a certain doctor or specialist*. *You know the story*. *But*, *if SCOPE had a centralized resource pool*, *those barriers to access could be removed*. *It would be even more seamless for the patient and the PCPs*. *Like*, *if a psychiatrist has capacity*, *why not send them someone on a waitlist? It is an equity issue that where you live impacts what services you get*.*”—PCP Lead*

### Challenges and threats to SCOPE-MH

Interview participants identified several challenges to the uptake and implementation of the SCOPE-MH program, as well as threats to SCOPE-MH sustainability. These included limited integration with existing hospital infrastructure and continuity of staff, physician funding and compensation, and challenges related to electronic health record interoperability.

At some sites, the SCOPE-MH team is fully embedded within the hospital infrastructure with staff seconded on a part-time basis to support the needs of the program. In other sites, staff were specifically hired to fill needs with the program. For those sites that hired new staff for their SCOPE-MH team, team members described an impact on the level of experience these hired staff had as the positions were contracted and typically attracted more junior health professionals.

“*In general it’s been very hard to recruit [staff]*, *and especially someone with any sort of mental health experience*. *When they hear that the position is temporary*, *they don’t want it*. *This means we tend to get new graduates*. *We actually took a risk this year and hired someone on a six month contract even though we didn’t know if there would be money*. *We took a risk because we really wanted to do this*. *We felt that there’s a need*.*”—Program Manager*

Without knowing whether funding was secured for the following year, or due to delays in receiving the funding, some sites were required to continue to hire staff on these rotating contracts. The lack of sustainable funding resulted in hire loss and inability to transfer the experience acquired with the program to the new recruits. In this situation, the role of senior or pilot sites mentoring the newer ones becomes key to maintain the institutional memory of the program.

Another challenge identified was around physician compensation. Under the current system in Ontario, physicians are paid on a per client basis with many restrictions on collaboration [14]. For example, PCPs are only permitted to bill for up to four teleconferences per year, per client. As a consequence, complex cases that require frequent case management and interprofessional collaboration may not be fully addressed or followed-up. Equally, immediate communication by phone from a psychiatrist to a PCP is not billable if it occurs on the same day the specialist has seen the patient. Adding to these limitations is that collaboration between a physician and an allied health provider is not billable unless another physician or a second allied health provider is present. Lastly, there is great disparity in the ability to bill in the acute-care setting compared to the inconsistent and often low stipends and rates available to psychiatrists doing outpatient or community-based work.

“*When it comes to e-consults*, *I mean*, *unless it is something very important where I would ask a patient to meet with me*, *I don’t spend a lot of time on it*. *E-consults are really underpaid*.*”—Psychiatrist*

The conjunction of all these regulations around physician compensation in the current system discourages junior psychiatrists from collaborative care and general outpatient care at large as it is not as profitable as inpatient care and often requires more administrative and record keeping effort that is not compensated. Without efficient and fair means of billing for consultation, including e-consultation, collaboration between PCPs and specialists like psychiatrists is threatened. This represents a structural paradox by which physicians are not compensated for work that is known to decrease patient care costs. Further, in the absence of competitive compensation, psychiatrists will continue to privilege private practice and connections to specialized mental health care will be restricted to acute-care settings.

Lastly, many sites reported difficulties with compatibility or interoperability of electronic health record systems.

“*I think from the system perspective*, *it would be ideal if we had a common [electronic health record] where everyone could access it*. *It may be dreaming a bit*, *but if something like that were available*, *I think you would save not only time but resources and it would help expedite referrals*.*”—PCP Lead*

PCPs and hospitals in Ontario are free to use any health record system so long as it is compliant with Canadian personal health information protection laws. This contributes to an excess of systems in use, with hospitals generally favouring more complex systems while PCPs tend to prefer more user-friendly, inexpensive electronic records. In the absence of compatibility between these systems, users resort to fax machines to communicate. This is a long-standing and well-documented challenge globally and one that is unlikely to be addressed in the near future [15, 16]. This is because there is no requirement for hospitals or PCPs to use common record systems in Canada, and despite existing within a publicly funded health system, the companies that create these health record systems are private, for-profit entities who, by design, create systems that are not compatible with one another. Until this challenge is addressed, even the most innovative and flexible of models of care will be limited.

## Discussion

Patients with mental health care needs are often high users of healthcare services with PCPs serving as these patients’ first point of contact with the healthcare system [17]. High-quality care for individuals living with this complexity requires functional and effective working relationships between PCPs, specialists, and other health professionals and is known to reduce the risk of acute episodes that lead to emergency room visits, hospitalization, readmission, and other poor health outcomes [18–21]. However, these relationships may be difficult to establish in the absence of mutuality, governance, and information systems [21, 22]. The SCOPE program and more specifically, the SCOPE-MH program, increase these needed linkages between unaffiliated PCP practices, hospitals, and community-based resources to address known challenges and fill gaps in the delivery of comprehensive mental health care in the primary care and community setting.

The SCOPE-MH model shares several key components with existing mental health collaborative care models. This includes an emphasis on interprofessional collaboration, care coordination, and facilitated access to mental health specialists as a means of advancing mental health care [23–25]. Further, in both the SCOPE-MH model and existing mental health collaborative care models, it is understood that co-localized care is a vital component of these models as it facilitates in-depth, patient-centered, care cooperation all while enhancing the expertise of the team through reciprocal learning [23]. With increasing uptake of virtual modalities in healthcare delivery, including in primary care and mental healthcare delivery, there are opportunities to enhance co-localized care by extending its reach beyond physical location, improving coordination and communication between providers across settings and sectors, and offering more patient-centric approaches. However, while technology can complement co-localized care, there is an imperative for careful planning, technological investments, and adaptation to ever-evolving healthcare policies and practices.

Through this qualitative improvement initiative, it was also found that barriers to implementing this care model and program sustainability were similar to those reported in the literature. This included difficulties with information sharing, limited staffing and training, and challenges with funding and reimbursement–all emphasizing the need for securing enhanced support systems, governance, and long-term funding [26, 27]. While all of these collaborative care models share elements, a distinguishing feature of the SCOPE-MH model is its particular focus on adaptability and equity within each individual community. Findings from this quality improvement initiative highlight the need for continued flexibility within these collaborative care models as well as the need for tailoring of services based on local community assets and needs.

### Next steps

The SCOPE-MH program is an innovative approach to enhancing the quality-of-care delivery, particularly in the context of mental health support for PCPs. Through this quality improvement initiative, the accessibility, adaptability, and flexibility of the program, leading to care that is both community tailored and personalized was highlighted. Further, this evaluation has illuminated the dynamic nature of the SCOPE-MH program, showcasing the adaptability of the model to the unique needs and assets of each community. The flexibility inherent in the design of the program allowed each site to adapt the program, including staffing composition, levels of psychiatric services, and program foci, to specific community needs even with the same fiscal resources. The successful application of this flexibility demonstrates that a one-size-fits-all approach is not conducive to optimizing patient care, is not responsive to the local context, and is not what healthcare providers find most meaningful in their practice. Building on these findings, and the challenges identified, two recommendations may be posed.

First, there is a need to advocate for sustainable funding and staffing to support initiatives like the SCOPE-MH program. This could include exploring funding models that encourage the retention of more experienced staff and the ability to offer long-term employment opportunities, rather than relying on short-term contracts. This may be done through ongoing mentorship and knowledge transfer between mature and newer SCOPE-MH sites to maintain institutional memory and expertise or the opportunity to explore more centralized resources to ensure equity across the entire program with site assets shared more broadly.

Second, and in order to enhance communication between PCPs and psychiatrists, a clear mechanism or pathway for consultation, that is sufficiently compensated, should be explored. This could include a model for consultation, one-off communication, or more robust collaboration for complex patient needs. Importantly, our findings underscore the necessity of avoiding a reliance on altruism and instead, advocating for a system that compensates psychiatrists, especially those doing work in the community, compensating adequately for their time and expertise. Furthermore, to successfully implement and sustain models of care such as SCOPE-MH, physicians must be engaged in the administrative aspects of the program. Providing stipends that liberates their clinical time ensures that they can actively contribute to developing these services without compromising the financial stability of their clinics.

### Limitations

While this quality improvement project captured various aspects of the SCOPE-MH program, including its development and implementation, it did not explicitly measure the effectiveness or impact of the program specific to patient outcomes or health improvements. There is a need for future analysis of, for example, patient-reported outcomes to assess how the program can impact patient mental health, treatment adherence, or overall health outcomes. Second, the evaluation drew from interviews with team members across all SCOPE-MH sites, providing diverse perspectives of the program, including its adoption, adaption, and future. However, participants included self-identified interdisciplinary team members, potentially leading to a sample that favoured those who were more involved, supportive, or knowledgeable about the program. This approach may have overlooked perspectives or experiences of individuals who are less engaged with the program, those who are critical of its implementation, or those who have transitioned away from the program. Lastly, this evaluation project presents a snapshot of the SCOPE-MH program at a specific point in time, detailing its development and challenges. However, a longitudinal analysis tracking the program’s progress over time could provide a deeper insight into its evaluation, sustained impact, and the resolution of any challenges faced during implementation. This longitudinal view could offer a more comprehensive understanding of how the program can be adapted to address emerging needs over time.

## Conclusion

The development and implementation of the SCOPE-MH program across diverse sites reflect its adaptability, effectiveness, and potential for long-term sustainability. Challenges related to communication, staffing, physician compensation and funding continuity must be addressed to ensure continued success, scalability, and optimized comprehensive mental health care.

This quality improvement initiative provides valuable insights for healthcare organizations looking to develop similar integrated mental health programs. It underscores the significance of tailoring interventions to local contexts, fostering strong healthcare professional relationships, and maintaining flexibility in program design. Ongoing evaluation and refinement will be essential as the program evolves to ensure its enduring impact on patient care and mental health support in underserved communities.

## References

[1] Lockhart E, Hawker G, Ivers N, O’Brien T, Mukerji G, Pariser P, et al. Engaging primary care physicians in care coordination for patients with complex medical conditions. Canadian Family Physician. 2019;65(4):e155–62. 30979773 PMC6467654

[2] Pariser P, O’Brien T, Kavanagh J, Stanaitis I, Salkovitch M, Rybak M. Seamless Care Optimizing the Patient Experience (SCOPE)—Co-designing the scaffolding for primary care integration. International Journal of Integrated Care. 2019;19:372.

[3] Gutkin C. Supporting Canada’s family physicians: The public has spoken, is anybody listening? Canadian Family Physician. 2006;52(4):548–7.

[4] Mental Health Commission of Canada. Advancing collaborative mental health care in primary care settings: A national quality framework with recommended measures 2022 [Available from: https://mentalhealthcommission.ca/resource/advancing-collaborative-mental-health-care/.

[5] Nundy S, Cooper LA, Mate KS. The quintuple aim for health care improvement: A new imperative to advance health equity. JAMA. 2022;327(6):521–2. doi: 10.1001/jama.2021.25181 35061006

[6] Marshall E, Miller L, Moritz L. Challenges and impacts from wait times for specialist care identified by primary care providers: Results from the MAAP study cross-sectional survey. Healthcare Management Forum. 2023;36(5). doi: 10.1177/08404704231182671 37415463 PMC10448708

[7] Advancing Integrated Mental Health Solutions. Collaborative care n.d. [Available from: https://aims.uw.edu/collaborative-care.

[8] Archer J, Bower P, Gilbody S, Lovell K, Richards D, Gask L, et al. Collaborative care for depression and anxiety problems. Cochrane Database of Systematic Reviews. 2012;10. doi: 10.1002/14651858.CD006525.pub2 23076925 PMC11627142

[9] VanderWielen L, Gilchrist E, Nowels M, Petterson S, Rust G, Miller B. Not near enough: Racial and ethnic disparities in access to nearby behavioral health care and primary care. Journal of Health Care for the Poor and Underserved. 2015;26(3):1032–47. doi: 10.1353/hpu.2015.0083 26320931 PMC4962556

[10] Reilly K, Kennedy S, Porter G, Estabrooks P. Comparing, contrasting, and integrating dissemination and implementation outcomes included in the RE-AIM and Implementation Outcomes frameworks. Frontiers in Public Health. 2020;8. 32984239 10.3389/fpubh.2020.00430 PMC7492593

[11] O’Brien B, Harris I, Beckman T, Reed D, Cook D. Standards for reporting qualitative research: A synthesis of recommendations. Academic Medicine. 2014;89(9):1245–51. doi: 10.1097/ACM.0000000000000388 24979285

[12] Itchhaporia D. The evolution of the quintuple aim. Journal of the American College of Cardiology. 2021;78(22):2262–4. 34823665 10.1016/j.jacc.2021.10.018 PMC8608191

[13] Harden S, Gaglio B, Shoup J, Kinney K, Johnson S, Brito F, et al. Fidelity to and comparative results across behavioral interventions evaluated through the RE-AIM framework: A systematic review. Systematic Reviews. 2015;4(1). doi: 10.1186/s13643-015-0141-0 26547687 PMC4637141

[14] Ontario Ministry of Health and Long-Term Care. Ontario Health Insurance Plan: Schedule of benefits and fees 2023 [Available from: https://www.health.gov.on.ca/en/pro/programs/ohip/sob/.

[15] Holen-Rabbersvik E, Thygesen E, Eikebrokk T, Fensli R, Slettebø A. Barriers to exchanging healthcare information in inter-municipal healthcare services: A qualitative case study. BMC Medical Informatics and Decision Making. 2018;18(92). doi: 10.1186/s12911-018-0701-z 30404630 PMC6223094

[16] Aller M, Vargas I, Coderch J, Vázquez M. Doctors’ opinion on the contribution of coordination mechanisms to improving clinical coordination between primary and outpatient secondary care in the Catalan national health system. BMC Health Services Research. 2017;17. 29273045 10.1186/s12913-017-2690-5 PMC5741878

[17] Smetanin P, Khan M, Ahmad S, Briante C, Stiff D. The life and economic impact of major mental illnesses in Canada. 2015.

[18] Kringos D, Boerma W, Hutchinson A, van der Zee J, Groenewegen P. The breadth of primary care: A systematic literature review of its core dimensions. BMC Health Services Research. 2010;10(65). doi: 10.1186/1472-6963-10-65 20226084 PMC2848652

[19] Huntley A, Lasserson D, Wye L, Morris R, Checkland K, England H, et al. Which features of primary care affect unscheduled secondary care use? A systematic review. BMJ Open. 2014;4:e004746. doi: 10.1136/bmjopen-2013-004746 24860000 PMC4039790

[20] Busby J, Purdy S, Hollingworth W. How do population, general practice, and hospital factors influence ambulatory care sensitive admissions: A cross sectional study. BMC Family Practice. 2017;18(67). doi: 10.1186/s12875-017-0638-9 28545412 PMC5445441

[21] Bodenheimer T, Ghorob A, Willard-Grace R, Grumbach K. The 10 building blocks of high-performing primary care. Annals of Family Medicine. 2014;12(2):166–71. doi: 10.1370/afm.1616 24615313 PMC3948764

[22] Wagner E. Chronic disease management: What will it take to improve care for chronic illness? Effective Clinical Practice. 1998;1(1). 10345255

[23] Rugkåsa J, Tveit O, Berteig J, Hussain A, Ruud T. Collaborative care for mental health: A qualitative study of the experiences of patients and health professionals. BMC Health Services Research. 2020;20:844. doi: 10.1186/s12913-020-05691-8 32907559 PMC7487713

[24] Reist C, Petiwala I, Latimer J, Raffaelli S, Chiang M, Eisenberg D, et al. Collaborative mental health care: A narrative review. Medicine (Baltimore). 2022;101(52). doi: 10.1097/MD.0000000000032554 36595989 PMC9803502

[25] Kroenke K, Unutzer J. Closing the false divide: Sustainable approaches to integrating mental health services into primary care. Journal of General Internal Medicine. 2017;32:404–10. doi: 10.1007/s11606-016-3967-9 28243873 PMC5377893

[26] Goodrich D, Kilbourne A, Nord K, Bauer M. Mental health collaborative care and its role in primary care settings. Current Psychiatry Reports. 2013;15. doi: 10.1007/s11920-013-0383-2 23881714 PMC3759986

[27] Castillo E, Ijadi-Maghsoodi R, Shadravan S, …, Wells K. Community interventions to promote mental health and social equity. Current Psychiatry Reports. 2019;21(35). doi: 10.1007/s11920-019-1017-0 30927093 PMC6440941

